# Spatial–temporal patterns of fish trophic guilds in a freshwater river wetland ecosystem of northeastern China

**DOI:** 10.1002/ece3.11711

**Published:** 2024-07-18

**Authors:** Xu Sun, Kai Wang, Ge Zhang, Han Ren, Hongxian Yu

**Affiliations:** ^1^ Key Laboratory of Applied Biology and Aquaculture of Northern Fishes in Liaoning Province, College of Fisheries and Life Science Dalian Ocean University Dalian Liaoning China; ^2^ Northeast Institute of Geography and Agroecology Chinese Academy of Sciences Changchun Jilin China; ^3^ University of Chinese Academy of Sciences Beijing China; ^4^ Department of Ecology Hebei University of Environmental Engineering Qinhuangdao Hebei China; ^5^ College of Life and Environmental Science Wenzhou University Wenzhou Zhejiang China; ^6^ Wetland Biodiversity Conservation and Research Center, College of Wildlife and Protected Area Northeast Forestry University Harbin Heilongjiang China

**Keywords:** environmental filters, fish, longitudinal gradients, Muling River basin, trophic guilds

## Abstract

Muling River, situated amidst cultivated lands in Heilongjiang Province, northeastern China, has long been subjected to sand‐digging activities, resulting in severe damage to its riverbed. However, little research has been conducted on the impact of this disturbance on the status of fish community structure and trophic guilds in this river. In this study, environmental factors, fish community structure, and fish trophic guild biomass distribution patterns from the Muling River basin were investigated among seasons (spring, summer, and autumn) and sections (upper, middle, and lower stream) in 2015 and 2017. During the six sampling times periods, 46 species of five orders and 12 families of fish were classified into seven trophic guilds. Fish species number and biomass were higher upper reaches of the watershed. The insectivores (16.26%), phytoplanktivores (10.09%), benthivores (40.17%), and omnivores (11.86%) were the dominant trophic guilds. We found that fish trophic guilds biomass and environmental factors such as transparency, water depth, pH value, total phosphorus, and chemical oxygen demand were highest in the upper section compared to other sections. Variation partitioning revealed that fish trophic guilds biomass was influenced more by environmental factors (61.2%), followed by section (0.7%) and season (0.1%). Partial RDA ordination showed that fish trophic guilds were positively correlated with water depth and transparency, while negative with turbidity. This study underscores the importance of considering trophic guilds of freshwater fishes to inform management strategies in regions experiencing significant environmental change.

## Graphical abstract

Partial RDA analysis variation partitioning results show that fish trophic guilds biomass could be explained more by environmental factors, followed by section, and season was the smallest.

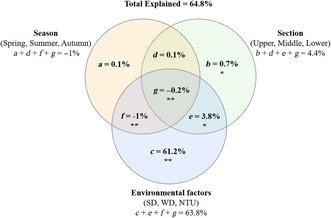

## 1 INTRODUCTION

Fish serve as crucial indicators for monitoring the health of freshwater aquatic ecosystems and are integral components of the aquatic food web (Persson et al., 2007; Whitfield, 2010). Global climate change is one of the primary factors leading to alterations in aquatic environments and the disruption of fish community structures. (Dala‐Corte et al., 2020; Ghisi et al., 2022). The growth of fish is influenced by numerous ecological interactions, including predation, competition, and parasitism, as well as environmental factors such as water velocity, nutrients, temperature, and light availability (Dala‐Corte et al., 2020; Kail et al., 2015). The structure and composition of fish communities reflect the broader ecological conditions of aquatic ecosystems and therefore may be used for monitoring water quality (Beaugrand et al., 2000; Rashleigh, 2004). Freshwater fish are vital components of aquatic ecosystems, playing key roles in ecosystem maintenance by providing food sources for humans and delivering socioeconomic and cultural services. (Chen et al., 2022; Mondal & Bhat, 2021).

A river ecosystem is a complex, open, dynamic, nonequilibrium, and nonlinear system (Jaehnig et al., 2015; Jiang, Chen, et al., 2018), where the community structure and functions are integrated with the watershed (Vannote et al., 1980). External factors influence the physical and hydrochemical characteristics of the river, including runoff, channel morphology, substrate type, water quality, and sediment properties (Shi et al., 2016). Simultaneously, the river ecosystem is susceptible to influences from its surrounding areas, including the effects of local human activities on water ecosystems and changes in other ecosystems (Zhu et al., 2013). Moreover, rivers serve as vital and dynamic ecological factors within numerous terrestrial ecosystems (Yu et al., 2012). The threat to freshwater organisms from environmental change and human activities is escalating (Zhang et al., 2022). Human activities have altered the physical and chemical conditions, as well as ecological processes in rivers, resulting in shifts in species composition and fish community abundance (Allan, 2004). Various environmental factors including hydrology, flow pattern, and energy input, exhibit significant spatial heterogeneity, resulting in alterations to the species composition and quantitative characteristics of stream fish communities (Wang et al., 2001). Analyzing the community construction mechanism across environmental gradients or spatial scales is beneficial as it serves as a key indicator of organism community. (Alcacio et al., 2020; Tolonen et al., 2017).

Most studies on river function ecosystems rely on the traditional species classification (Arthur et al., 2010; Chowdhury et al., 2011; Petty et al., 2005). However, recent studies indicate that ecosystem function primarily hinges on functional traits, encompassing the spatial and temporal distribution patterns and abundance of these functional traits (Elliott & Quintino, 2010; Li, Gou, Wang, Ma, et al., 2021). Analyzing functional characteristics can anticipate interference effects before species disappearance or extinction, offering the potential for early warning systems (Villéger et al., 2017). Trophic guilds are sensitive to environmental changes and play a crucial role in studying the relationship between biological communities and ecosystem function (Becker et al., 2010; Ren et al., 2021). Trophic guilds are indicators for assessing environmental change and ecological response, reflecting the functions of each ecosystem component (Gonzalez et al., 2021). Trophic guilds represent structural units within fish populations in specific water bodies, forming the basis of the ecosystem's nutrient cycling and ecological stability (Lobry et al., 2008; Mangadze et al., 2016). Trophic guilds, defined by biological characteristics, are closely linked to ecosystem processes, which are crucial for understanding ecosystem and community functions (Jiang, Liu, et al., 2018; Nash et al., 2021). The specific traits of trophic guilds are intricately connected to the environment, enabling a more direct reflection of ecological processes within aquatic communities. This facilitates a better comprehension of aquatic ecosystems and their community structure.

Muling River is one of the important rivers in Heilongjiang Province in Northeast China. In recent decades, the river has been seriously damaged by residents' continuous discharge of domestic sewage, industrial wastewater, and agricultural practices on both sides of the river. It leads to the depletion of fish fauna and the increasingly serious imbalance of aquatic ecosystems (Bacigalupi et al., 2021). With the aggravation of agricultural nonpoint source pollution, industrial discharge pollution, and urban life pollution in the Muling River basin, the water quality is deteriorating, which has harmed the production and life of local people. However, little study was done on the impact of this disturbance on the status of fish community structure and diversity in this river. In this study, we investigated fish assemblages and environmental factors, revealing the relationship between fish functional trophic guilds and environmental factors. This has important theoretical and practical significance for clarifying the characteristics of fish community structure, aquatic organism protection, water environment health, and water ecological health evaluation, which is associated with human disturbance.

## 2 MATERIALS AND METHODS

### 2.1 Study area

**FIGURE 1:**
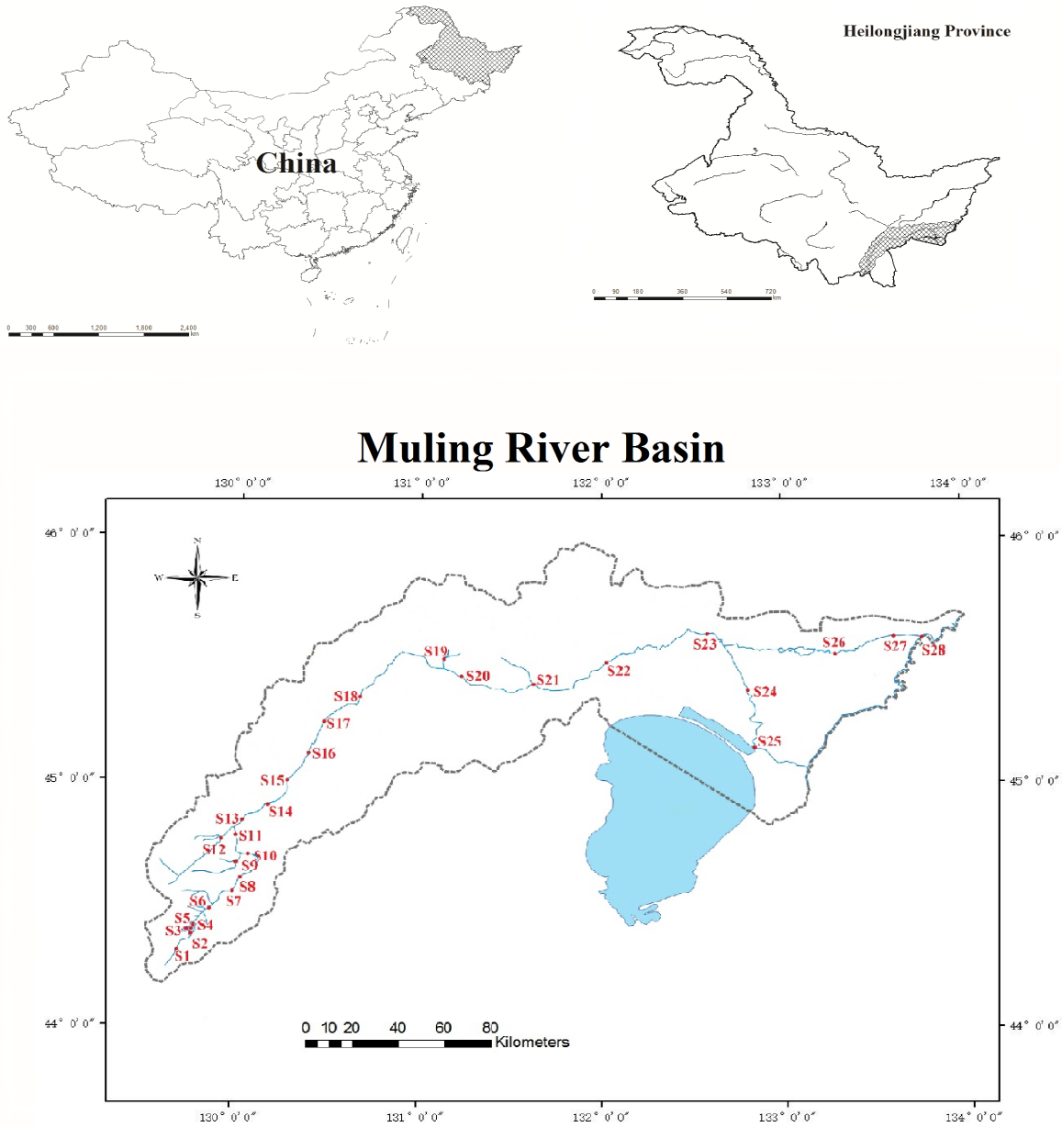
Map of sampling sites in the Muling River basin.

Muling River traverses through Muling, Jixi, Jidong, Mishan, and Hulin cities (Figure 1). It spans a length of 834 km, with its basin covering an area of 18,427 km^2^, predominantly surrounded by farmlands (Sun & Wang, 2021). The upper reaches experience a temperate continental climate characterized by hot, rainy summers, and long and cold winters. The upper reaches receive an average annual precipitation of 530 mm, primarily occurring from July to September. The middle reaches experience a temperate semi‐humid monsoon climate, with an average annual temperature of 3.1°C, ranging from −18 to 21°C. The region receives an annual precipitation of 522 mm with a frost‐free period lasting 149 days. Precipitation serves as the primary source of water supply in the middle and lower reaches of the Muling River basin, supplemented by surface water and paddy infiltration water.

### 2.2 Data sampling

According to the local climate and ecological environment of the Muling River Basin, six sampling campaigns were conducted in May (Spring), July (Summer), and September (Autumn) in both 2015 and 2017. A total of 28 sampling sites were established throughout the basin (Figure 1), with each sampling site being sampled three times. Sites S1 to S18 were situated in the upper stream, sites S19 to S23 in the middle stream, and sites S24 to S28 in the lower stream. Furthermore, we utilized the 2015 and 2017 year averages as our dataset to mitigate errors arising from sampling in this study.

Fish samples catch per unit effort (CPUE) were mainly obtained using 1 m × 40 m gill‐net (mesh 3–7 cm) and 2 m ground bamboo cage (mesh 1 cm), and three replicates were deployed 24 h in each sampling site with the assistance of fishermen, which test that fish biomass (g) differs between sites and seasons/year. Additionally, we interviewed local villagers to investigate the status of fish resources. Biological measurements, including the standard length and weight of the fish, were taken in situ. Unidentified samples were stored in 75% alcohol and transported back to the laboratory for further identification.

According to the requirements of water and wastewater monitoring methods (Wei, 2002), the environmental factors measured including transparency (SD, m), water depth (WD, m), electrical conductivity (EC, mS/cm), dissolved oxygen (DO, mg/L), pH value (pH), water temperature (T, °C), total nitrogen (TN, mg/L), total phosphorus (TP, mg/L), ammonia nitrogen (${\mathrm{NH}}_{4}^{+}$–N, mg/L), nitrate nitrogen (${\mathrm{NO}}_{3}^{-}$–N, mg/L), chemical oxygen demand (COD_Mn_, mg/L), oxidation–reduction potential (ORP, mV), biochemical oxygen demand (BOD_5_, mg/L), turbidity (NTU), and flow velocity (FV, m/s) were measured.

### 2.3 Data analysis

In the laboratory, we referred to the keys of Zhang and He (1993), Zhang (1995), Zhu (1995), and Chen (1998) for species identification. The fish biomass is calculated by the weight of fish in the sampling unit. The fish trophic guilds were divided according to the fish's food habits, close relatives, and feeding organ structure (Ding & Liu, 2011). These seven types were found: aquatic plant trophic guild (herbivores, HE), aquatic insect trophic guild (insectivores, IN), phytoplanktivores trophic guild (phytoplanktivores, PH), zooplanktivores trophic guild (zooplanktivores, ZO), benthic animal trophic guild (benthivores, BE), omnivores trophic guild (omnivores, OM), piscivores trophic guild (piscivores, PI).

We employed the Shapiro–Wilk test to assess the normality of all variables, obtaining a *p* value smaller than .05, which means our data were not normally distributed. Subsequently, we conducted a two‐factor permutational multivariate analysis of variance (PERMANOVA, 999 permutations) to examine the impact of sampling sections and periods on overall environmental factors and fish trophic guilds biomass, as well as their interaction. To explore how sections and periods influence on each element of environment and fish trophic guilds. Then, we utilized a two‐way analysis of “adonis2” function and pairwise. adonis's multiple comparisons to examine the statistical significance of environmental factors and fish trophic guilds biomass in different periods (Spring, Summer, and Autumn) and section sites (Upper stream, Middle stream, and Lower stream). All these analyses were carried out using R 4.1.2 (R Core Team, 2021) with the “vegan” (Oksanen et al., 2022) and “pairwiseAdonis” (Martinez Arbizu, 2020) packages.

Using Canoco 5.0 software, we analyzed the impact of environmental factors, period, and section impact on fish trophic guilds biomass. To ensure normal distribution, the fish trophic guilds biomass data were transformed using the formula log_10(*x* + 1) (Sun et al., 2019). Firstly, we conducted a forward selection filter out variables that meet the requirements, and three environment factors were selected (WD, SD, and NTU). Then, the detrended corresponding analysis (DCA) of the largest gradient length of the four axes was 2.168 which is lower than 3. Therefore, the linear ordination method of the redundancy analysis (RDA) was selected to reveal the relationship. In addition, Monte Carlo simulations with 499 permutations were used to test the significance of the environmental factors in explaining the biomass of fish trophic guilds data in the RDA.

## 3 RESULTS

### 3.1 Fish species composition and biomass distribution

**FIGURE 2:**
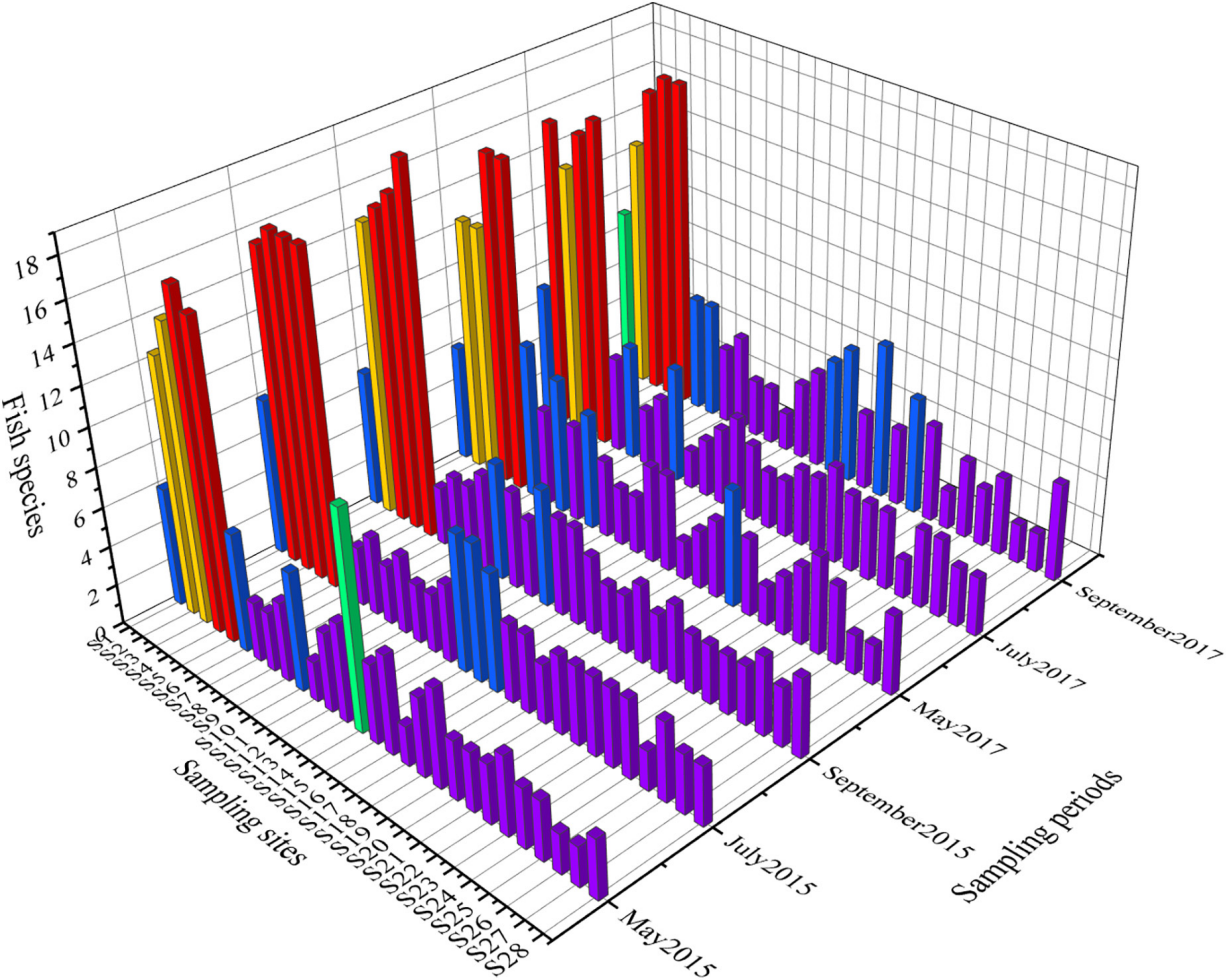
Distribution of fish species in the Muling River basin. Different colors meaning five quantile groups of 100–80% (red), 79–60% (yellow), 59–40% (green), 39–20% (blue), and 19–0% (purple).

We identified a total of 46 fish species belonging to 12 families across five orders during the survey, as detailed in Table S1. The biological characteristics of fish catches are provided in Table S2. Among these species, 32 species (69.57%) belong to 25 genera within two families in the order Cypriniformes, while five species (10.87%) belong to five genera of three families in the order Salmoniformes. Additionally, four species (8.7%) from four genera within four families were classified under the order Perciformes, and another four species (8.7%) from three genera within two families were categorized under the order Siluriformes. Finally, one species (2.17%) from a single genus within one family falls under the order Petromyzoniformes. The observed number of fish species varied among individual sampling sites in the Muling River basin, ranging from 2 to 19 (Figure 2). The representative fish species of the Muling River basin are shown in Figure S1.

**FIGURE 3:**
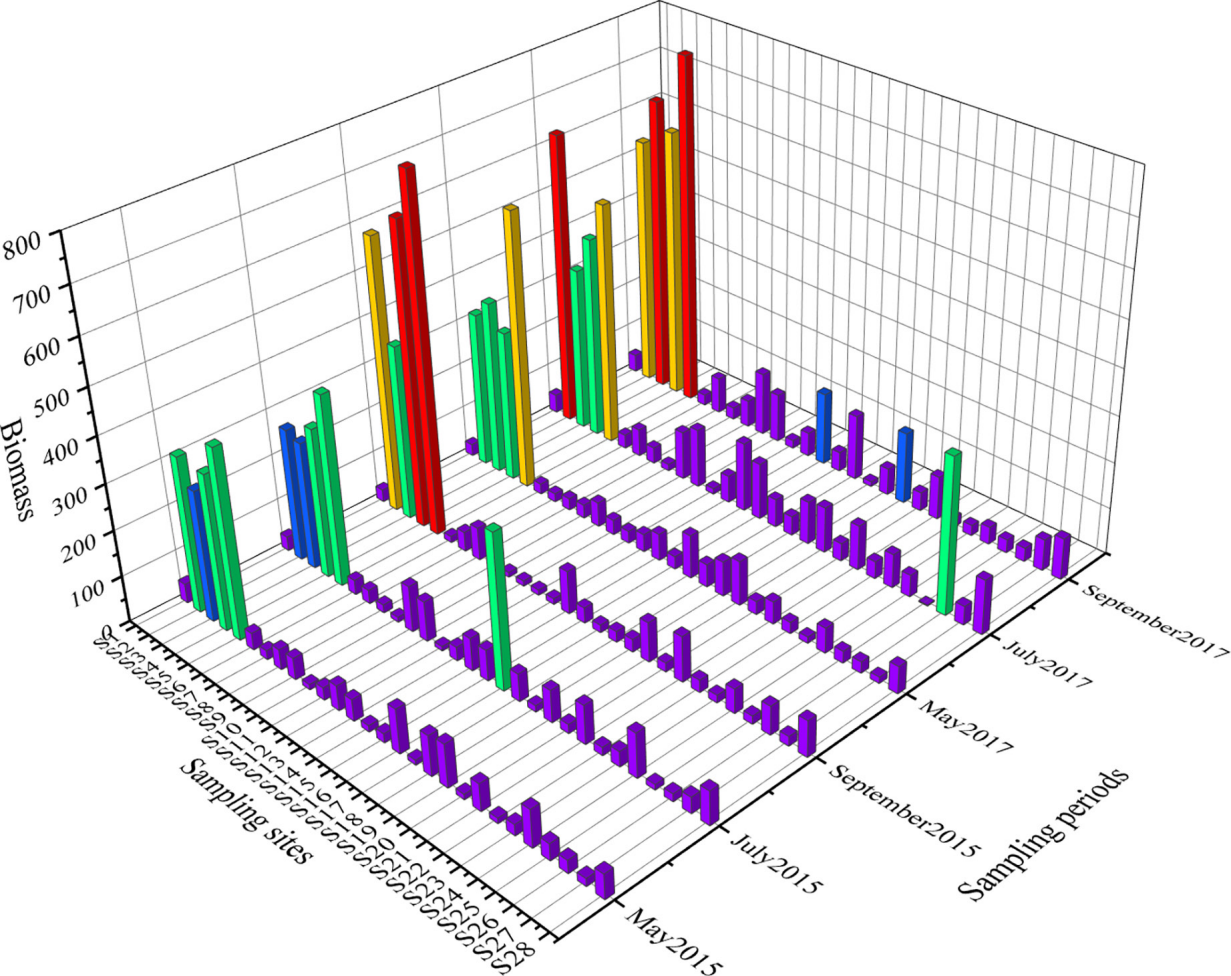
Distribution of fish biomass (g) in the Muling River basin. Different colors meaning five quantile groups of 100–80% (red), 79–60% (yellow), 59–40% (green), 39–20% (blue), and 19–0% (purple).

The fish biomass in the Muling River basin ranged from 8.22 to 770.36 g (Figure 3). In the sampling period of May 2015, S5 observed the highest biomass (401.69 g), followed by S2 with 333.53 g, while S14 observed the lowest biomass (11.8 g). In July, S5 observed the highest biomass (395.39 g), followed by S4 with 317.2 g, while S26 observed the lowest biomass (11.4 g). In September, S5 biomass was the highest with 770.36 g, followed by S4 with 651.04 g, while S12 was observed the lowest (11.67 g). While in the sampling period of May 2017, S5 observed the highest biomass (605 g), followed by S3 with 374.18 g, while S27 observed the lowest biomass (11.3 g). In July, S2 observed the highest biomass (641.19 g), followed by S5 with 532.34 g, while S12 observed the lowest biomass (12.84 g). In September, S5 observed the highest biomass (768.33 g), followed by S3 with 649.11 g, while S17 observed the lowest biomass (8.22 g).

### 3.2 Dominant fish trophic guilds characteristics

Seven fish trophic guilds were grouped in the Muling River Basin (Table 1). The piscivores trophic guild was most fish species (14 species, 30.43%), followed by the omnivores trophic guild (eight species, 17.39%), aquatic insect trophic guild and benthic animal trophic guild (seven species, 15.22%), phytoplanktivores trophic guild (three species, 6.52%), and zooplanktivores trophic guild was the lowest (only one species, 2.17%). The dominant fish trophic guilds of the Muling River basin were aquatic insect trophic guild (16.26%), phytoplanktivores trophic guild (10.09%), benthic animal trophic guild (40.17%), and omnivores trophic guild (11.86%). Characteristics of changes in the relative biomass of fish trophic guilds sampled in six replicates are shown in Figure S2. Moreover, during sampling sections, IN and BE trophic guilds dominated the whole basin. PH was only dominant in the upper stream, while OM was dominant in the upper and lower streams of the Muling River. While, during sampling periods, IN, BE, and OM dominated the whole season, and PH dominated in summer and autumn. The spatio‐temporal distribution of dominant fish trophic guilds is shown in Figure S3.

**TABLE 1:** Fish trophic guilds and biomass percentage of Muling River basin.

Trophic guilds	Species	Sections (%)			Periods (%)			Total (%)
		Upper	Middle	Lower	Spring	Summer	Autumn	
HE	Grass carp (*Ctenopharyngodon idellus*), Bail minnow (*Phoxinus phoxinus*), Lagowsky minnow (*Phoxinus lagowskii*)	9.01	4.72	4.12	8.85	9.19	8.76	8.20
IN	Lake minnow (*Phoxinus percnurus*), Rice loach (*Lefua costata*), Northern weatherfish (*Misgurnus bipartitus*)	**15.25**	**14.76**	**26.56**	**22.55**	**18.48**	**13.27**	**16.26**
PH	Cheskanowsky's minnow (*Phoxinus czekanowskii*), Amur gudgeon (*Rostrogobio amurensisi*), Sliver carp (*Hypophthalmichthys molitrix*)	**11.54**	2.90	3.76	9.19	**10.65**	**12.70**	**10.09**
ZO	Bighead carp (*Aristichthys nobilis*)	5.70	0.00	0.00	4.77	3.75	6.68	4.70
BE	Common sawbelly (*Hemiculter leucisclus*), Wheat head fish (*Pseudorasbora parva*), Chinese false gudgeon (*Abbottina rivularis*), Chinese Lizard gudgeon (*Saurogobio dabryi*), Luther's spined loach (*Cobitis lutheri*), Granoc's spined loach (*Cobitis granoci*), Amur Weatherfish (*Misgurnus mohoity*)	**37.56**	**63.48**	**42.27**	**43.67**	**43.74**	**43.83**	**40.17**
OM	Common carp (*Cyprinus carpio*), Prussian carp (*Carassius auratus gibelio*)	**11.15**	9.37	**20.44**	**10.17**	**13.41**	**14.57**	**11.86**
PI	Amur catfish (*Silurus asotus*), Chinese sleeper (*Perccottus glehni*), Asiatic brook lamprey (*Lampetra reissneri*)	9.79	4.78	2.85	9.65	9.96	8.96	8.72

*Note*: Bold indicates dominant trophic guilds (relative biomass percentage > 10%). Sections: S1–S18‐Upper, S19–S23‐Middle, and S24–S28‐Lower; Periods: May (Spring), July (Summer), and September (Autumn), in 2015 and 2017, respectively. The abbreviations of variables followed Table S3.

### 3.3 Sampling sections and periods influence on environmental factors and fish trophic guilds

We first detected the sampling sections and periods effects on overall environmental factors and fish trophic guilds biomass (Table 2). We found that environmental factors showed significant differences during sampling sections (*p* < .001), periods (*p* < .001), and their interaction (*p* < .01). While fish trophic guilds only showed significant differences in sections (*p* < .05), we examined the effects of different sections and seasons on environmental factors. We found that environmental factors of upper‐stream section were significantly different than other sections (*p*.adjusted < .01), and showed significant differences during all seasons (Spring, Summer, and Autumn) (*p*.adjusted < .05). Whereas, no overall difference was found in different sampling sections and periods (*p*.adjusted > .05) of fish trophic guilds (Tables 2 and 3).

**TABLE 2:** Effects of sampling sections, sampling periods, and their interaction on environmental factors and fish trophic guilds biomass examined by Two‐way permutational multivariate analysis of variance (PERMANOVA, 999 permutations) with fish trophic guilds composition as the random term.

	Item	*R* ^2^	*F*	*p*
Environmental factors	Sections	**.283**	**22.69**	<.001***
	Periods	**.172**	**13.789**	<.001***
	Sections × Periods	**.078**	**3.111**	.008**
Trophic guilds	Sections	**.051**	**2.119**	.028*
	Periods	.01	0.395	.955
	Sections × Periods	.03	0.621	.917

*Note*: Sections: S1–S18‐Upper, S19–S23‐Middle, and S24–S28‐Lower; Periods: May (Spring), July (Summer), and September (Autumn) in 2015 and 2017, respectively. Sections × Periods means the interaction of sampling sections and sampling periods. Bold values indicate a significant difference: *p < .05, **p < .01, ***p < .001.

**TABLE 3:** PERANOVA test for testing environmental factors and trophic guilds in different sampling sections and periods for analysis of variance and multiple comparisons.

	Environmental factors				Trophic guilds			
	*F*	*R* ^*2*^	*p*.value	*p*.adjusted	*F*	*R* ^*2*^	*p*.value	*p*.adjusted
**Sections**								
Upper vs. Middle	30.899	.316	.001	**.003**	2.805	.04	.022	.066
Upper vs. Lower	10.807	.139	.001	**.003**	2.018	.029	.072	.216
Middle vs. Lower	2.917	.094	.066	.198	1.018	.035	.413	1
**Periods**								
Spring vs. Summer	13.38	.199	.001	**.003**	0.545	.01	.736	1
Spring vs. Autumn	7.047	.115	.001	**.003**	0.475	.009	.82	1
Summer vs. Autumn	5.632	.094	.007	**.021**	0.168	.003	.98	1

*Note*: The *p*.adjusted represents the corrected *p*.value, where a value >.05 indicates that the result from *R* is not statistically significant. The sections are categorized as follows: S1–S18 for the Upper section, S19–S23 for the Middle section, and S24–S28 for the Lower section. The periods correspond to May for Spring, July for Summer, and September for Autumn, occurring in 2015 and 2017, respectively.

From Table 4, we found that the effect of sampling periods on environmental factors was much more significant than that sampling sections. This difference in environmental factors can be seen from the F value: the sampling periods' F value ranged from 0.604 to 317.254, which was higher than that of the sampling sections (0.103 to 36.258). Most of the environmental factors exhibited significant differences in different sampling periods. Table 5 reveals that certain environmental factors (SD, WD, PH, TP, and COD_Mn_) were highest in the upper section, while others were lowest. Most environment factors peaked in autumn (SD, EC, DO, PH, ${\mathrm{NO}}_{3}^{-}$–N, and BOD_5_) and summer (WD, T, TN, COD_Mn_, ORP, NTU, and FV), whereas only two factors (${\mathrm{NH}}_{4}^{+}$–N and TP) peaked in spring.

**TABLE 4:** Statistical significance of two‐way ANOVA was assessed using the adonis2 test, a nonparametric test, with 15 environmental factors and seven fish trophic guilds biomass

		Sections		Periods		Sections × periods	
		*F*	*p*	*F*	*p*	*F*	*p*
Environmental factors	SD	**5.486**	.004**	2.249	.127	0.039	.998
	WD	2.227	.109	0.604	.531	0.035	.999
	EC	**36.145**	.001**	**15.433**	.001**	2.086	.091
	DO	0.131	.892	**18.704**	.001**	0.207	.931
	pH	**12.040**	.002**	**21.349**	.001**	**9.035**	.001**
	T	**7.990**	.003**	**317.254**	.001**	**5.661**	.001**
	TN	2.714	.071	**39.373**	.001**	1.877	.130
	TP	**3.433**	.032*	**10.874**	.002**	2.410	.077
	${\mathrm{NH}}_{4}^{+}$–N	**8.838**	.001**	0.673	.648	2.476	.091
	${\mathrm{NO}}_{3}^{-}$–N	**36.258**	.001**	**25.261**	.001**	**5.297**	.001**
	COD_Mn_	**26.514**	.001**	**25.261**	.001**	2.627	.051
	ORP	**6.113**	.005**	**4.558**	.022*	2.005	.106
	BOD_5_	1.333	.275	**8.340**	.002**	1.108	.347
	NTU	**20.410**	.001**	**8.746**	.001**	**5.559**	.005**
	FV	3.141	.058	**4.647**	.007**	1.014	.418
Trophic guilds	HE	**3.752**	.031*	0.159	.856	0.022	1
	IN	1.305	.271	0.216	.841	0.280	.859
	PH	**4.272**	.023*	0.591	.571	0.256	.907
	ZO	**3.274**	.042*	0.577	.584	0.160	.962
	BE	1.758	.192	0.293	.759	0.047	.997
	OM	1.174	.322	0.700	.510	0.402	.822
	PI	**4.793**	.014*	0.140	.876	0.057	.993

*Note*: Sections: S1–S18‐Upper, S19–S23‐Middle, and S24–S28‐Lower; Periods: May (Spring), July (Summer), and September (Autumn) in 2015 and 2017, respectively. Sections × Periods means the interaction of sampling sections and sampling periods. Bold values indicate a significant difference: *p < .05, **p < .01. The abbreviations of variables followed Table S3.

**TABLE 5:** Environmental factors and fish trophic guilds biomass (g) changes in different sampling sections and periods (Mean ± SE).

		Sections			Periods		
		Upper	Middle	Lower	Spring	Summer	Autumn
Environmental factors	SD	0.42 ± 0.03b	0.18 ± 0.02a	0.34 ± 0.05b	0.39 ± 0.03b	0.28 ± 0.04a	0.41 ± 0.04b
	WD	2.10 ± 0.59a	1.53 ± 0.07a	1.83 ± 0.10a	2.97 ± 0.58a	3.63 ± 0.78a	3.10 ± 0.67a
	EC	0.12 ± 0.01a	0.23 ± 0.01c	0.17 ± 0.01b	0.13 ± 0.01a	0.13 ± 0.01a	0.19 ± 0.01b
	DO	7.56 ± 0.26a	7.81 ± 0.58a	7.21 ± 0.56a	7.70 ± 0.24b	6.23 ± 0.43a	8.70 ± 0.38c
	pH	7.90 ± 0.06b	7.37 ± 0.19a	7.54 ± 0.19a	7.60 ± 0.10a	7.47 ± 0.14a	8.15 ± 0.04b
	T	15.05 ± 0.53a	15.86 ± 1.21a	17.13 ± 1.18a	14.50 ± 0.35b	22.13 ± 0.39c	10.06 ± 0.53a
	TN	2.45 ± 0.16a	2.94 ± 0.19a	2.49 ± 0.29a	1.63 ± 0.12a	3.36 ± 0.23c	2.64 ± 0.20b
	TP	0.48 ± 0.03a	0.38 ± 0.04a	0.37 ± 0.03a	0.52 ± 0.04b	0.49 ± 0.04b	0.32 ± 0.02a
	${\mathrm{NH}}_{4}^{+}$–N	0.22 ± 0.01a	0.32 ± 0.03b	0.67 ± 0.25b	0.37 ± 0.13a	0.25 ± 0.02a	0.34 ± 0.05a
	${\mathrm{NO}}_{3}^{-}$–N	1.35 ± 0.14a	3.76 ± 0.76b	1.55 ± 0.41a	0.81 ± 0.06a	1.99 ± 0.27b	2.64 ± 0.46b
	COD_Mn_	4.30 ± 0.07b	3.62 ± 0.16a	3.61 ± 0.13a	3.85 ± 0.09a	4.49 ± 0.09b	3.82 ± 0.13a
	ORP	50.87 ± 1.74a	51.47 ± 3.95a	63.26 ± 3.27b	47.45 ± 2.56a	56.07 ± 2.90b	56.05 ± 2.03b
	BOD_5_	1.81 ± 0.10a	2.07 ± 0.16a	1.64 ± 0.11a	1.72 ± 0.10a	1.49 ± 0.08a	2.27 ± 0.17c
	NTU	39.30 ± 3.48a	186.84 ± 32.84b	158.66 ± 42.96b	38.91 ± 3.30a	141.68 ± 28.80b	80.29 ± 12.68b
	FV	0.12 ± 0.01b	0.21 ± 0.03a	0.11 ± 0.02b	0.15 ± 0.02b	0.18 ± 0.03b	0.08 ± 0.01a
Trophic guilds	HE	15.33 ± 1.99a	3.89 ± 1.11b	3.78 ± 1.09b	9.95 ± 2.31a	13.38 ± 2.60a	12.52 ± 2.49a
	IN	24.24 ± 3.36a	10.95 ± 2.67a	18.25 ± 5.31a	22.13 ± 5.15a	23.33 ± 4.26a	18.11 ± 3.17a
	PH	21.09 ± 2.72a	2.39 ± 0.68b	3.65 ± 1.95b	11.16 ± 2.26a	15.51 ± 2.82a	20.08 ± 4.61a
	ZO	12.64 ± 1.85a	0.00 ± 0.00b	0.00 ± 0.00b	6.88 ± 1.73a	6.66 ± 1.65a	12.53 ± 3.10a
	BE	52.31 ± 6.60a	31.40 ± 4.42a	23.23 ± 3.85a	36.62 ± 6.26a	45.18 ± 7.52a	47.87 ± 9.11a
	OM	21.17 ± 2.88a	6.32 ± 1.44a	16.85 ± 6.22a	12.03 ± 2.47a	18.59 ± 3.53a	22.44 ± 4.62a
	PI	15.73 ± 1.97a	3.23 ± 0.71b	2.76 ± 1.06b	10.11 ± 2.01a	13.48 ± 2.73a	12.23 ± 2.56a

*Note*: Mean values with different letters indicate significant differences (*p* < .05), while values with the same letters indicate no significant differences (*p* > .05) across various sampling periods and periods. Sections: S1–S18‐Upper, S19–S23‐Middle, and S24–S28‐Lower; Periods: May (Spring), July (Summer), and September (Autumn) in 2015 and 2017, respectively. The abbreviations of variables followed Table S3.

Regarding fish trophic guild biomass (Table 5), significant differences were observed among different sections (*p* < .05). However, there was no significant change in fish trophic guild biomass across different periods (*p* > .05). The highest biomass of fish trophic guilds was found in the upper section, ranging from 15.33 to 52.31 g.

### 3.4 Variance partitioning of fish trophic guilds biomass

**FIGURE 4:**
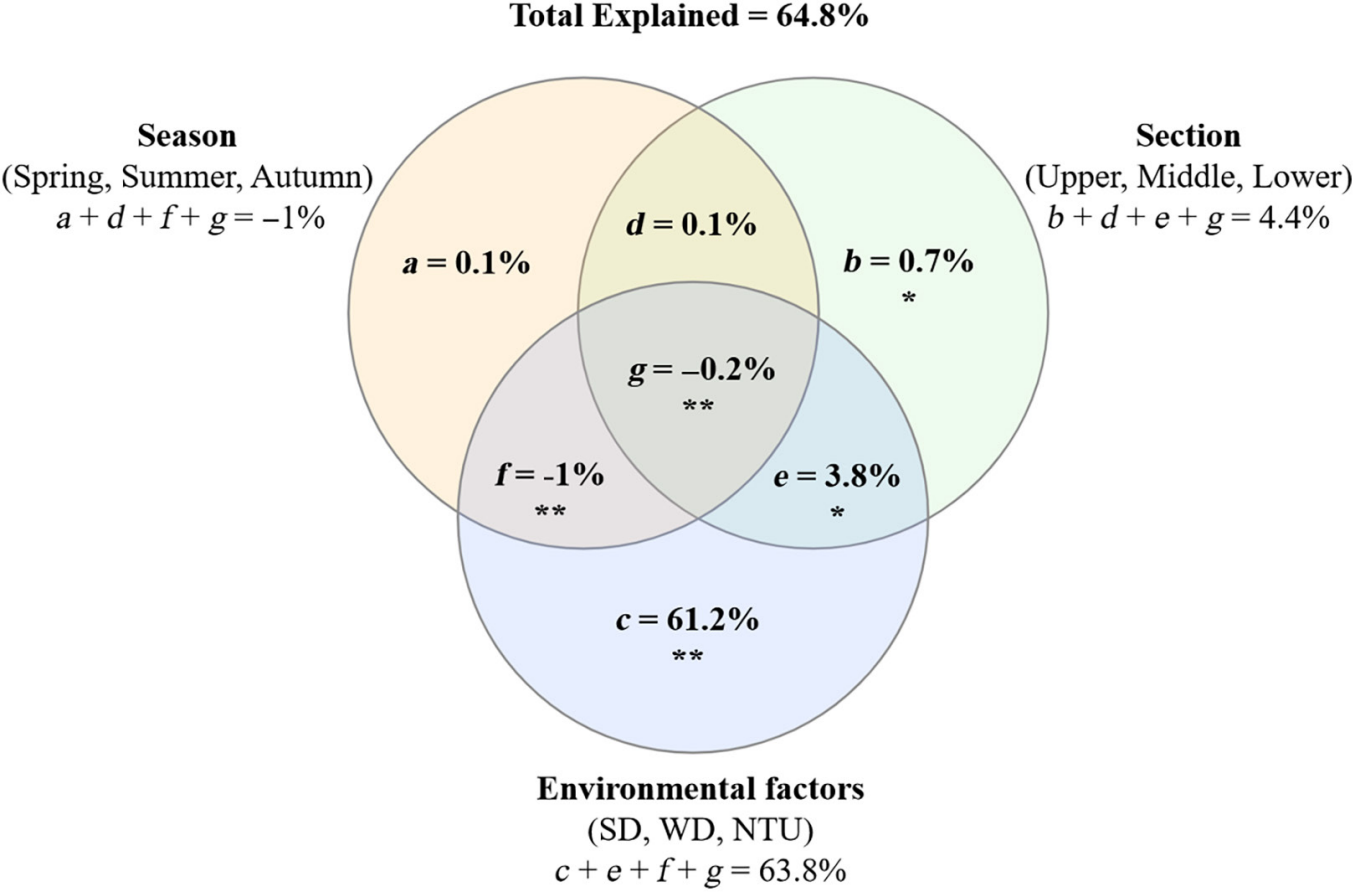
Variation partitioning results for three groups in partial RDA analysis “Var‐part‐3groups‐Conditional‐effects‐tested” in biomass. Season: Spring, Summer, and Autumn; Section: Upper, Middle, and Lower stream; Environmental factors: Transparency (SD, m), water depth (WD, m), and turbidity (NTU).

The variance partitioning results showed that three groups (Section, Season, and Environmental factors) could explain 64.8% of fish trophic guilds biomass variations. Fish trophic guilds biomass could be explained more by environmental factors, followed by section, and season was the smallest. When explanatory variables were divided into three groups, section alone explained 0.7%, and environmental factors alone explained 61.2%, but season alone explanation was not significant (Figure 4).

### 3.5 Redundancy ordination between fish trophic guilds biomass and environmental factors

**FIGURE 5:**
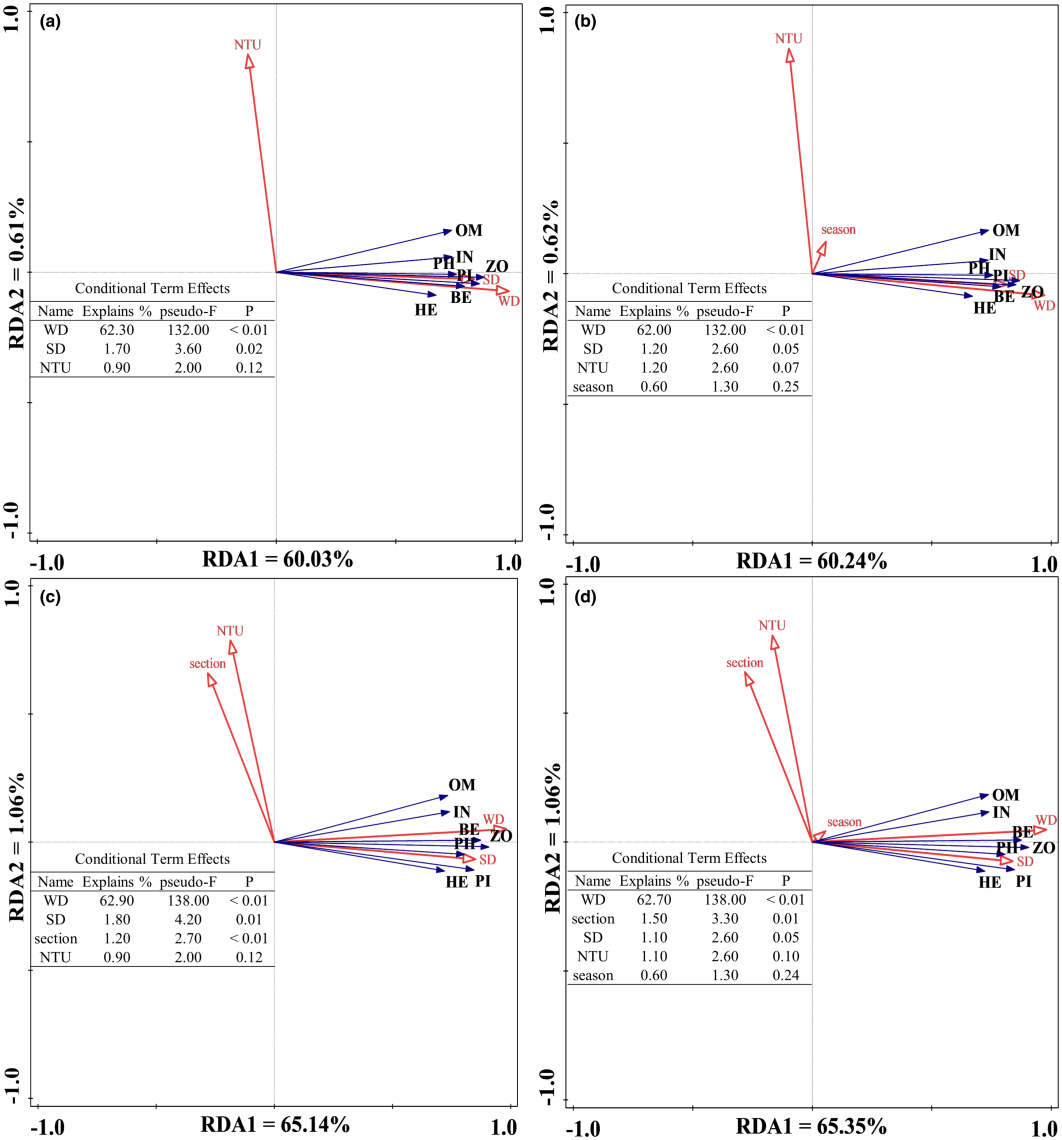
RDA ordination results between fish trophic guilds and environmental factors in pooled data. Partial RDA ordination after excluding section and season impact (a); partial RDA ordination after excluding section impact (b); partial RDA ordination after excluding sampling season impact (c); RDA ordination of fish trophic guilds, environmental factors, season, and section (d). Season includes Spring, Summer, and Autumn; Section contains Upper, Middle, and Lower streams. The abbreviations of variables followed Table S3.

After excluding section and season, section, and season parameters, respectively, the RDA partial ordination manifested the fish trophic guilds biomass were positively related to WD and SD, while slightly negatively associated with NTU (Figure 5a–c). The highest explanatory power both were WD at 62.30%, 62.00%, and 62.9%, followed by SD (1.70%, 1.20%, and 1.80%). When excluding section parameters, all fish trophic guilds were tiny positively with season (0.60%, *p* = .25) (Figure 5b); while excluding season parameters, they were negatively related to section (1.20%, *p* < .01) (Figure 5c). Similar tendencies were found in the RDA ordination, in which the highest explanation power was WD at 62.70%, followed by the sampling section at 1.50%, and SD at 1.10% (Figure 5d).

Three environmental variables (SD, WD, NTU) were filtered out by using forward selection same as variance partitioning, which Spearman correlation analysis results further confirming that SD, WD, and NTU were significantly correlated with fish trophic guilds than other environmental factors (Figure S4).

## 4 DISCUSSION

This study is the first to examine the spatial–temporal gradient variation in fish trophic guilds in the Muling River basin. Our results indicate significant differences in environmental factors across section, season, and their interaction. However, fish trophic guilds biomass was more strongly influenced by environmental factors, followed by section, with season having the least impact. RDA ordination results found that increasing transparency and water depth could significantly enhance the fish community.

### 4.1 Fish fauna composition

The fish fauna is shaped by the interactions between different fish populations and their long‐term influence (Epur, 2009; Li & Xie, 2004; Zeng & Zhou, 2012). Based on fish origin (Zhao et al., 2007), distribution (Liu et al., 2011), and living habits (Gushchin & Fall, 2012), the fish fauna in Muling River Basin consists of five faunal groups: (1) the Upper Tertiary faunal group, formed in the early Tertiary period and located in the northern temperate zone of the Northern Hemisphere, (2) the Northern Plain faunal group, formed in the plain areas of the northern sub cold zone of the Northern Hemisphere, (3) the Northern Mountainous Areas faunal group, formed in the foothills of the sub cold zone in the northern hemisphere, (4) the River Plains faunal group, species formed in the Tertiary in the plains of eastern China, and (5) the Subtropical Plain faunal group, mostly hypoxia‐tolerant species (Sun et al., 2019).

Based on Li (1981) classification of freshwater fish fauna in China, the Muling River basin is located in the transitional sub‐region of Heilongjiang Province within the Palaearctic realm, where northern and southern faunal characteristics blend. Unlike the broader fish fauna of Heilongjiang Province, the basin lacks cold‐resistant species typically associated with Arctic freshwater fauna. The Muling River basin features a temperate continental climate that is not conducive to the survival of certain species. In comparison with the nearby water systems, the Wusuli River hosts 42 species of fish (Ren, 1994), while 20 of these species are also found in the Muling River, suggesting that Muling River is the largest tributary on the left bank of the Wusuli River. The fish fauna of the Ussuri River, while closely related to that of the Muling River, also exhibits clear differences.

### 4.2 Responses of fish trophic guilds to section and season

During the sampling period, dominant fish species in Muling River constituted 67.60% of the total fish population (Sun et al., 2019). These species form the core of the community structure and play a critical role in shaping its structure and function (Bonaldo et al., 2017). The study identified seven fish trophic guilds, with four dominant groups: insectivores, phytoplanktivores, benthivores, and omnivores (Table 1). Insectivores and benthivores trophic guilds dominated across all seasons through the 2 years study period. Muling River supports a significant population of aquatic insects and benthic organisms, offering abundant food sources for insectivores and benthivores trophic guilds (Sun et al., 2021). Liu et al. (2016) observed that phosphorus salt significantly impacted benthos and indirectly influenced the trophic guilds of benthic‐feeding fish, particularly Luther's spined loach. The phytoplanktivores trophic guild dominated in the autumn of 2015 and the summer and autumn of 2017. This dominance might result from increased rainfall, which boosted surface runoff and indirectly raised nitrogen and phosphorus concentrations in rivers, thereby increasing the phytoplankton population in autumn (Cao et al., 2018). Additionally, the omnivorous trophic guild dominated during both the summer and autumn through the 2‐year sampling period. Common carp and Prussian carp emerged as dominant species following the growth season of aquatic organisms, indicating their adaptation to the habitat environment.

The Muling River basin, situated in the agricultural wetland ecological area of Sanjiang Plain, undergoes large‐scale cultivation in spring (Luo et al., 2022). Our investigation revealed that the total biomass of phytoplankton peaked in spring and hit its lowest point in summer, aligning with findings from the Daning River findings (Zhu et al., 2013). Fasham et al. (1990) and Shumka et al. (2018) suggested that increased nutrient concentrations result in a higher abundance of planktonic organisms, serving as the primary driver for dynamic changes in phytoplankton community structure (Tilman et al., 1982). Chen et al. (2019) found that nitrogen can enter the water through fish secretion and excretion. Algae subsequently absorb these nitrogenous nutrients, enriching the water and providing ample nutrients for plankton growth. This process fosters the proliferation of dominant species, including Granoc's spined loach (*Cobitis granoei*), Heilongjiang Weatherfish (*Misgurnus mohoity*), and Wheat head fish (*Pseudorasbora parva*). Moreover, it could enhance the food sources for carnivorous fish like Chinese sleeper (*Perccottus glehni*). However, only the Chinese sleeper occupied a dominant position in the PI group. Amur catfish is a significant catch in the basin, but overfishing has resulted in dwindling numbers. The Asiatic brook lamprey was exclusively found in the upstream area, where its larvae and juveniles typically inhabited the sandy bottom and were buried in the sand. Human activities, such as digging sand in the middle and the downstream regions, destroyed its habitat. Consequently, neither of the aforementioned fish species emerged as the dominant in these areas.

In addition to natural factors, human activities have emerged as significant disruptors of fish habitats (Li et al., 2012). The upper stream of Muling River, nestled in mountainous terrain, boasts dense forest vegetation, pristine water bodies, and a well‐preserved ecological environment, harboring numerous small‐sized fish species. Sampling sites S1 to S5, situated in the Tuanjie Reservoir at the river's upper stream, experience minimal water pollution (Sun et al., 2019). In the upper stream, local villagers prioritize environmental protection, and maintaining excellent water quality. However, in the mid‐stream, the presence of numerous sand pits contributes to increased turbidity and reduced water transparency due to ongoing sand excavation activities (Corell et al., 2023). Furthermore, this area is densely populated, resulting in the discharge of domestic sewage and agricultural runoff, significantly degrading water quality and leading to a reduction in fish species diversity to only seven (Sun et al., 2019). Additionally, large‐scale sand excavation operations, including machinery activities and the accumulation of excavated sand, not only damage the river channel but also obstruct the upstream migration of downstream fish, further decreasing fish populations. Conversely, downstream reaches boast rich fish species diversity, as this area is closer to the estuary and receives fish migration from the Ussuri River, resulting in a higher abundance of fish species (Hellmrich et al., 2023).

### 4.3 Impacts of river environmental changes on fish resources

The Muling River basin is encircled by farmland, including crops, rice, and maize. Extensive sand dredging over time has significantly damaged the riverbed, leading to ecosystem instability (Li, Gou, Wang, La, & Liu, 2021; Xu et al., 2001). Pesticides and fertilizers used in agricultural fields accumulate nutrients, which are subsequently washed into the river through surface runoff following rainfall events (Xiao et al., 2016). The Muling River's water is characterized by high turbidity and sediment content, resulting in sediment accumulation at the river bottom. This phenomenon causes the riverbed to rise and diminishes its flood discharge capacity (Ishii et al., 2021). Extensive land degradation surrounding the river basin exacerbates the situation, leading to a decline in the river's soil conservation function, reduced water storage capacity following a rainstorm, limited regulatory capabilities, and increased instances of severe floodings (Jia et al., 2014; Wu et al., 2019).

Agricultural soils surrounding the Muling River basin contain quantities of pesticides and fertilizers. These substances are transported into the river via surface runoff following rainfall events (Cao et al., 2018; Wang, Cai, Xu, et al., 2011; Xiao et al., 2016). During the survey period, we observed consistent trends in TN concentrations in 2015 and 2017, with higher levels in summer compared to spring and autumn (Table 5). In July 2017, the maximum ${\mathrm{NH}}_{4}^{+}$–N concentration recorded was 2.731 mg/L, exceeding the threshold for Class IV water (>1.5 mg/L), rendering it unsuitable for human consumption (Yu et al., 2012). In September 2015, the ${\mathrm{NO}}_{3}^{-}$–N concentration was as low as 0.28 mg/L. However, by 2017, this concentration had surged, reaching a level 18 times higher than before (Sun et al., 2019). This increase could be attributed to heavy rainfall exacerbating surface runoff, thereby introducing more nutrients into the rivers (Otero et al., 2011). Additionally, we observed phenomena related to nitrogen (N) and phosphorus (P) phenomena limitation (Hoffman et al., 2022; Rhea et al., 2021). Throughout 2015, N‐limitation was evident (TN:TP < 16) (Redfield, 1934), with the lowest value recorded in May 2017, indicating continued nitrogen restriction (TN:TP = 5.21). Subsequently, a rapid increase occurred, peaking in July of the same year (TN:TP = 17.97 > 16), signaling a shift to P‐limitation, with values remaining close to the critical threshold, reaching as high as 15.76 in the fall.

Currently, there are no large‐scale controlled water storage projects with extensive controlled areas along the mainstream of the Muling River. Fendou Reservoir, situated in the upper reaches of the Muling River, is characterized by mountainous terrain. The construction of the Fendou reservoir dam (at S11) in the mountainous region has had a significant impact on local microclimate (Wang, Cai, Tan, & Kong,  2011). Post‐dam construction, the rise in river water levels has facilitated plant growth and development in the valley area, thereby enhancing the overall functionality of the water body (Hui et al., 2014). Additionally, the decreased water flow rate has led to an accumulation of organic matter and nutrients (Wang, Cai, Xu, et al., 2011), fostering the growth and reproduction of plankton, benthic animals, and aquatic vascular plants, and subsequently increasing fish populations (Zhang et al., 2010, 2017). Regardless of the fish number, the fish species composition can undergo significant alterations. The construction of dams can introduce new species while causing the disappearance of previously documented ones due to habitat modifications (Lațiu et al., 2022). However, the detrimental effects of reservoir construction, such as alterations in water levels, hindrance to fish migration, and habitat fragmentation, warrant attention (Larsen et al., 2021; Liang et al., 2021; Pess et al., 2021).

### 4.4 Implications

At the basin level, our findings reveal that environmental factors especially WD and SD improvement could enhance the fish community. Thus, more monitoring management should be done to maintain the quality of the fresh water. A researcher in Amazonian implies that conservation of the local fisher community requires sufficient cover of forest cover, aquatic habitats, and floodplain vegetation (Arantes et al., 2018). Thus, not only environmental factors but also forest cover along the river should be taken into account, and riparian vegetation and larger area floodplains within this study should be included (Arantes et al., 2018). We should pay attention to large‐scale areas to include more environmental factors, such as land cover change (Arantes et al., 2018), climate change, floor terrain (Borland et al., 2021), and development of agriculture (Tockner & Stanford, 2002).

Second, considering significant differences in fish communities across low, middle, and high sections. Our results show higher fish trophic guilds biomass at the upper level, while lower fish trophic guilds biomass at the lower section. In different sites of fresh water, a study in the Bita River Basin found that the headwaters section contained higher fish diversity (López‐Delgado et al., 2020) because of small human influence and higher water quality. Different fish communities may reflect ecosystem ecological restoration (Legendre, 2014). On the one hand, more attention should be given to the spatial distribution of different fish communities. On the other hand, emphasizing efforts to enhance headwaters protection, is crucial for enhancing the fish community.

Finally, we have observed the disappearance of Lenok (*Brachymystax lenok*) for many years. This species was listed in the National Key Protected Wildlife List, recognized as a second‐level protected animal, and categorized as a vulnerable (VU) species in the Red List of Chinese Species. Historically, this fish population was only found in lower streams, and its presence was confirmed only through interviews and investigation conducted during the sampling periods (Table S1). Therefore, in addition to protecting and restoring the ecological environment of the basin, greater attention should be given to the rejuvenation of animal populations.

## 5 CONCLUSIONS

During the investigation period, 46 species of five orders and 12 families of fish were identified in the Muling River Basin, with biomass ranging from 8.22 to 770.36 g. Seven trophic guilds were divided, and the dominant trophic guilds were insectivores, phytoplanktivores, benthivores, and omnivores. RDA analysis showed that increasing transparency and water depth could significantly enhance fish trophic guilds, while the spatial factor (section) had a negative impact. In addition, environmental factors had a higher influence than spatial and seasonal factors in the local region. Our research findings will contribute to maintaining the stability of fish community structure, predicting dominant species, and assisting relevant technical personnel in quantifying river water quality through the monitoring of fish communities. In future fish protection efforts, greater attention should be given to protecting the basin environment and water quality, as well as selecting suitable sites for fish communities.

## AUTHOR CONTRIBUTIONS

**Xu Sun:** Formal analysis (lead); investigation (lead); methodology (lead); software (lead); writing – original draft (lead); writing – review and editing (equal). **Kai Wang:** Data curation (equal); formal analysis (equal); methodology (equal); software (equal); visualization (equal); writing – review and editing (equal). **Ge Zhang:** Investigation (equal); methodology (equal); supervision (equal); validation (equal). **Han Ren:** Formal analysis (equal); methodology (equal); software (equal); validation (equal); visualization (equal). **Hongxian Yu:** Conceptualization (equal); investigation (equal); methodology (equal); project administration (equal); resources (equal); supervision (equal); writing – review and editing (equal).

## CONFLICT OF INTEREST STATEMENT

The authors declare no conflict of interest in this study.

## Supporting information


Data S1.

## Data Availability

The data that support the findings of this study are openly available in Dataset at https://doi.org/10.5061/dryad.pc866t1tc, reference number not available.
