# Supplementary material for: Spatial–temporal patterns of fish trophic guilds in a freshwater river wetland ecosystem of northeastern China
Source: Ecol Evol. 2024 Jul 18;14(7):e11711. doi: 10.1002/ece3.11711 (PMC11257708; doi:10.1002/ece3.11711)
Supplement: Supplementary file 1 — Data S1. [file ECE3-14-e11711-s001.docx]

**Online supplement materials**

**Table S1** List of fish species, functional groups and geographical distribution in Muling River Basin. Trophic guilds: aquatic plant trophic guild (herbivores, HE), aquatic insect trophic guild (insectivores, IN), phytoplanktivores trophic guild (phytoplanktivores, PH), zooplanktivores trophic guild (zooplanktivores, ZO), benthic animal trophic guild (benthivores, BE), omnivores trophic guild (omnivores, OM), piscivores trophic guild (piscivores, PI).

| Order | Family | Genus | Species | Survey method | Trophic guild | Section | | | Season | | | 2015 | 2017 |
| --- | --- | --- | --- | --- | --- | --- | --- | --- | --- | --- | --- | --- | --- |
|  |  |  |  |  |  | upper | middle | lower | Spring | Summer | Autumn |  |  |
| Petromyzoniformes | Petromyzonidea | *Lampertra* | *Lampetra reissneri* | Sampling | PI | + |  | + | + | + | + | + | + |
| Salmoniformes | Salmonoidae | *Oncorhynchus* | *Oncorhynchus keta* | Interview & investigation | PI |  |  | + | + | + | + | + | + |
|  |  | *Hucho* | *Hucho taimen* | Interview & investigation | PI |  |  | + | + | + | + | + | + |
|  |  | *Brachymystax* | *Brachymystax lenok* | Interview & investigation | PI |  |  | + | + | + | + | + | + |
|  | Thymallidae | *Thymallus* | *Thymallus arcticus grubei* | Interview & investigation | IN | + |  |  | + | + | + | + | + |
|  | Esocidae | *Esox* | *Esox reicherti* | Interview & investigation | PI |  |  | + | + | + | + | + | + |
| Cypriniformes | Cyprinidae | *Opsariichthys* | *Opsariichthys bidens* | Interview & investigation | PI | + |  | + | + | + | + | + | + |
|  |  | *Mylopharyngodon* | *Mylopharyngodon piceus* | Interview & investigation | OM |  |  | + | + | + | + | + | + |
|  |  | *Ctenopharyngodon* | *Ctenopharyngodon idellus* | Sampling | HE | + |  | + | + | + | + | + | + |
|  |  | *Phoxinus* | *Phoxinus phoxinus* | Sampling | HE | + |  |  | + | + | + | + | + |
|  |  |  | *Phoxinus percnurus* | Sampling | IN | + |  | + | + | + | + | + | + |
|  |  |  | *Phoxinus czekanowskii* | Sampling | PH | + |  |  | + | + | + | + | + |
|  |  |  | *Phoxinus lagowskii* | Sampling | HE | + |  |  | + | + | + | + | + |
|  |  | *Leuciscus* | *Leuciscus waleckii* | Interview & investigation | OM |  |  | + | + | + | + | + | + |
|  |  | *Elopichthys* | *Elopichthys bambusa* | Interview & investigation | PI |  |  | + | + | + | + | + | + |
|  |  | *Hemiculter* | *Hemiculter leucisclus* | Sampling | BE | + | + |  | + | + | + | + | + |
|  |  | *Erythroculter* | *Erythroculter ilishaeformis* | Interview & investigation | PI |  |  | + | + | + | + | + | + |
|  |  |  | *Erythroculter mongolicus* | Interview & investigation | PI |  |  | + | + | + | + | + | + |
|  |  | *Parabramis* | *Parabramis pekinensis* | Interview & investigation | OM |  |  | + | + | + | + | + | + |
|  |  | *Megalobrama* | *Megalobrama amblycephala* | Interview & investigation | OM |  |  | + | + | + | + | + | + |
|  |  | *Xenocypris* | *Xenocypris argentea* | Interview & investigation | HE |  |  | + | + | + | + | + | + |
|  |  |  | *Xenocypris microlepis* | Interview & investigation | HE |  |  | + | + | + | + | + | + |
|  |  | *Rhodeus* | *Rhodeus sericeus* | Interview & investigation | HE |  |  | + | + | + | + | + | + |
|  |  | *Hemibarbus* | *Hemibarbus labeo* | Interview & investigation | IN |  |  | + | + | + | + | + | + |
|  |  | *Pseudorasbora* | *Pseudorasbora parva* | Sampling | BE | + | + | + | + | + | + | + | + |
|  |  | *Sarcocheilichthys* | *Sarcocheilichthys lacutris* | Interview & investigation | IN |  |  | + | + | + | + | + | + |
|  |  | *Abbottina* | *Abbottina rivularis* | Sampling | BE | + |  | + | + | + | + | + | + |
|  |  | *Rostrogobio* | *Rostrogobio amurensisi* | Sampling | PH | + | + |  | + | + | + | + | + |
|  |  | *Saurogobio* | *Saurogobio dabryi* | Sampling | BE | + |  | + | + | + | + | + | + |
|  |  | *Cyprinus* | *Cyprinus carpio* | Sampling | OM | + | + | + | + | + | + | + | + |
|  |  | *Carassius* | *Carassius auratus gibelio* | Sampling | OM | + | + | + | + | + | + | + | + |
|  |  | *Aristichthys* | *Aristichthys nobilis* | Sampling | ZO | + |  | + | + | + | + | + | + |
|  |  | *Hypophthalmichthys* | *Hypophthalmichthys molitrix* | Sampling | PH | + |  | + | + | + | + | + | + |
|  | Cobitidae | *Lefua* | *Lefua costata* | Sampling | IN | + |  |  | + | + | + | + | + |
|  |  | *Cobitis* | *Cobitis lutheri* | Sampling | BE | + |  |  | + | + | + | + | + |
|  |  |  | *Cobitis granoci* | Sampling | BE | + |  |  | + | + | + | + | + |
|  |  | *Misgurnus* | *Misgurnus mohoity* | Sampling | BE | + |  |  | + | + | + | + | + |
|  |  |  | *Misgurnus bipartitus* | Sampling | IN | + |  |  | + | + | + | + | + |
| Siluriformes | Suluridae | *Silurus* | *Silurus asotus* | Sampling | PI | + | + | + | + | + | + | + | + |
|  | Bagridae | *Pelteobagrus* | *Pelteobagrus fulvidraco* | Interview & investigation | OM | + |  | + | + | + | + | + | + |
|  |  |  | *Pelteobagrus nitidus* | Interview & investigation | IN | + |  | + | + | + | + | + | + |
|  |  | *Pseudobagrus* | *Pseudobagrus ussuriensis* | Interview & investigation | OM |  |  | + | + | + | + | + | + |
| Perciformes | Serranidae | *Siniperca* | *Siniperca chuatsi* | Interview & investigation | PI | + |  | + | + | + | + | + | + |
|  | Percidae | *Lucioperca* | *Lucioperca lucioperca* | Interview & investigation | PI |  |  | + | + | + | + | + | + |
|  | Eleotridae | *Perccottus* | *Perccottus glehni* | Sampling | PI | + | + | + | + | + | + | + | + |
|  | Channidae | *Channa* | *Channa argus* | Interview & investigation | PI |  |  | + | + | + | + | + | + |

**Table S2** Biological characteristics of fish catches.

| Species | Individual number | Total weight (g) | Average weight (g) | Weight range (g) | Average length (cm) | Length range (cm) |
| --- | --- | --- | --- | --- | --- | --- |
| Asiatic brook lamprey (*Lampetra reissneri*) | 139 | 887.09 | 6.38 | 2.86~9.69 | 15.41 | 11.70~19.34 |
| Grass carp (*Ctenopharyngodon idellus*) | 386 | 25243.17 | 65.40 | 12.56~98.64 | 15.72 | 10.34~23.65 |
| Bail minnow (*Phoxinus phoxinus*) | 258 | 1830.37 | 7.09 | 4.67~15.42 | 6.95 | 5.47~10.52 |
| Lake minnow (*Phoxinus percnurus*) | 876 | 3850.24 | 4.40 | 0.76~9.82 | 7.27 | 4.03~11.42 |
| Cheskanowsky’s minnow (*Phoxinus czekanowskii*) | 304 | 1902.64 | 6.26 | 1.52~8.64 | 4.95 | 3.54~8.67 |
| Lagowsky minnow (*Phoxinus lagowskii*) | 502 | 2011.74 | 4.01 | 0.82~8.43 | 5.98 | 3.76~9.43 |
| Common sawbelly (*Hemiculter leucisclus*) | 555 | 7031.85 | 12.67 | 7.62~20.85 | 10.45 | 6.35~13.68 |
| Wheat head fish (*Pseudorasbora parva*) | 491 | 1892.37 | 3.85 | 0.75~7.52 | 6.54 | 3.52~8.43 |
| Chinese false gudgeon (*Abbottina rivularis*) | 671 | 4665.28 | 6.95 | 0.62~14.82 | 6.33 | 4.04~9.35 |
| Amur gudgeon (*Rostrogobio amurensisi*) | 382 | 3161.50 | 8.28 | 0.98~28.64 | 6.31 | 3.84~13.46 |
| Chinese Lizard gudgeon (*Saurogobio dabryi*) | 1011 | 10814.95 | 10.70 | 0.74~19.85 | 7.88 | 2.55~18.43 |
| Common carp (*Cyprinus* *carpio*) | 1098 | 337347.61 | 307.24 | 63.42~1479.52 | 27.05 | 13.34~47.82 |
| Prussian carp (*Carassius auratus gibelio*) | 591 | 14751.25 | 24.96 | 5.46~187.42 | 10.21 | 4.87~18.72 |
| Bighead carp (*Aristichthys nobilis*) | 765 | 156225.98 | 204.22 | 28.75~2490.52 | 50.42 | 12.53~78.53 |
| Sliver carp (*Hypophthalmichthys molitrix*) | 749 | 785714.22 | 1049.02 | 14.82~1844.34 | 21.42 | 9.75~51.23 |
| Rice loach (*Lefua costata*) | 552 | 4502.93 | 8.16 | 1.21~23.57 | 8.88 | 5.43~15.43 |
| Luther’s spined loach (*Cobitis lutheri*) | 1320 | 10346.89 | 7.84 | 1.75~16.52 | 7.54 | 4.85~13.42 |
| Granoc’s spined loach (*Cobitis granoci*) | 1122 | 7147.14 | 6.37 | 1.42~13.64 | 6.76 | 4.25~12.54 |
| Amur Weatherfish (*Misgurnus mohoity*) | 724 | 6776.64 | 9.36 | 2.04~14.65 | 9.34 | 5.42~17.51 |
| Northern weatherfish (*Misgurnus bipartitus*) | 522 | 4489.2 | 8.60 | 2.84~17.85 | 9.39 | 6.75~16.81 |
| Amur catfish (*Silurus asotus*) | 723 | 22877.63 | 31.64 | 4.85~52.64 | 13.68 | 5.75~23.42 |
| Chinese sleeper (*Perccottus glehni*) | 355 | 3841.10 | 10.82 | 3.54~19.51 | 6.65 | 4.62~15.62 |

**Table S3** The abbreviations of environmental factors and fish trophic guilds used in all statistical analysis.

|  | Abbreviations | Variables |
| --- | --- | --- |
| Environmental factors | SD | Transparency |
|  | WD | Water depth |
|  | EC | Electrical conductivity |
|  | DO | Dissolved oxygen |
|  | pH | pH value |
|  | T | Water temperature |
|  | TN | Total nitrogen |
|  | TP | Total phosphorus |
|  | NH_4_^+^-N | Ammonia nitrogen |
|  | NO_3_^-^-N | Nitrate nitrogen |
|  | COD_Mn_ | Chemical oxygen demand |
|  | ORP | Oxidation-reduction potential |
|  | BOD_5_ | Biochemical oxygen demand |
|  | NTU | Turbidity |
|  | FV | Flow velocity |
| Trophic guilds | HE | Herbivores |
|  | IN | Insectivores |
|  | PH | Phytoplanktivores |
|  | ZO | Zooplanktivores |
|  | BE | Benthivores |
|  | OM | Omnivores |
|  | PI | Piscivores |

| 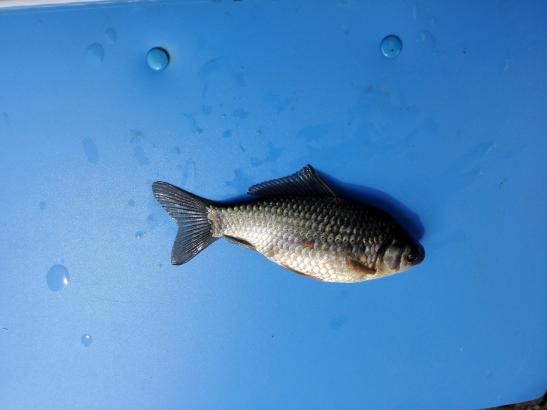  Prussian carp (*Carassius auratus gibelio*) | 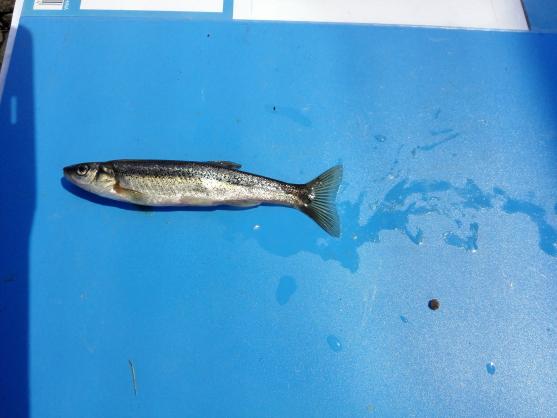  Lake minnow (*Phoxinus percnurus*) | 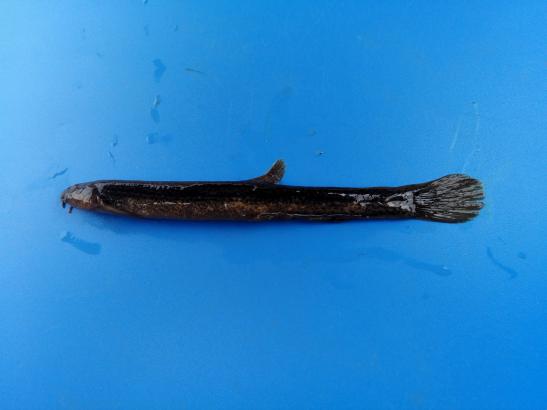  Northern weatherfish (*Misgurnus bipartitus*) |
| --- | --- | --- |
| 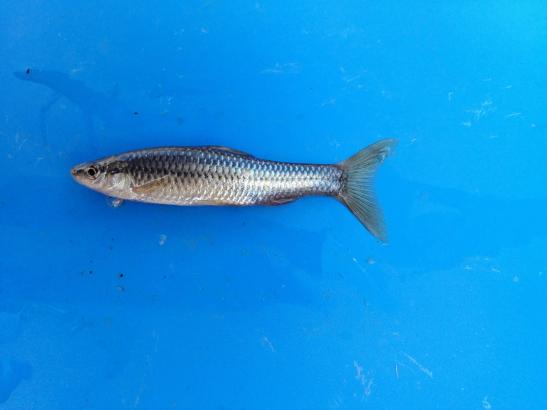  Wheat head fish (*Pseudorasbora parva*) | 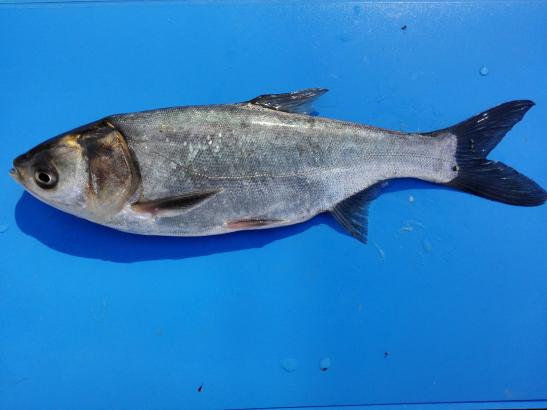  Sliver carp (*Hypophthalmichthys molitrix*) | 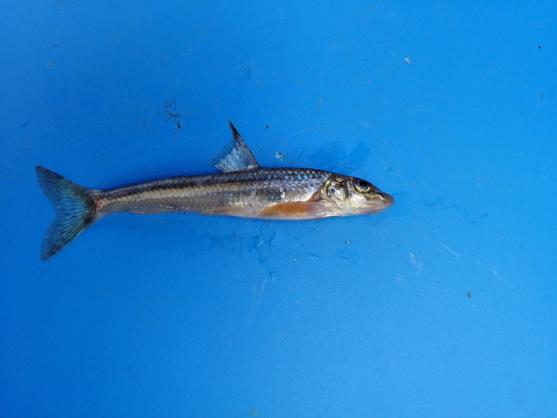  Chinese Lizard gudgeon (*Saurogobio dabryi*) |
| 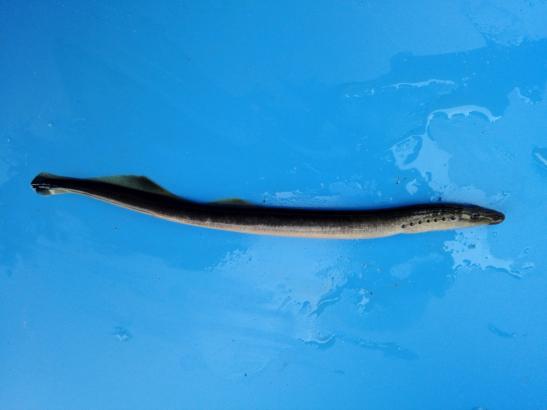  Asiatic brook lamprey (*Lampetra reissneri*) | 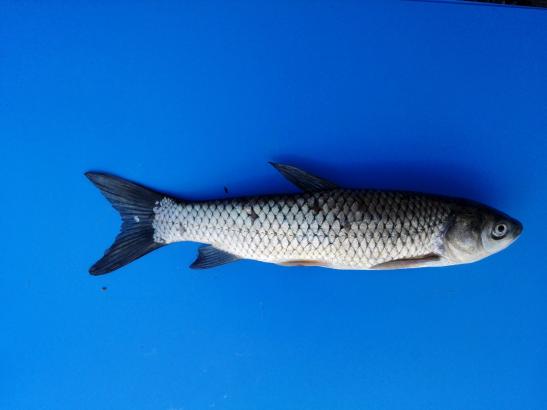  Grass carp (*Ctenopharyngodon idellus*) | 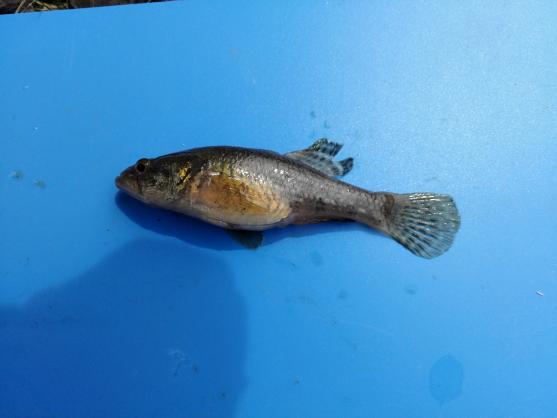  Chinese sleeper (*Perccottus glehni*) |

**Fig. S1** Representative fish species of Muling River basin.


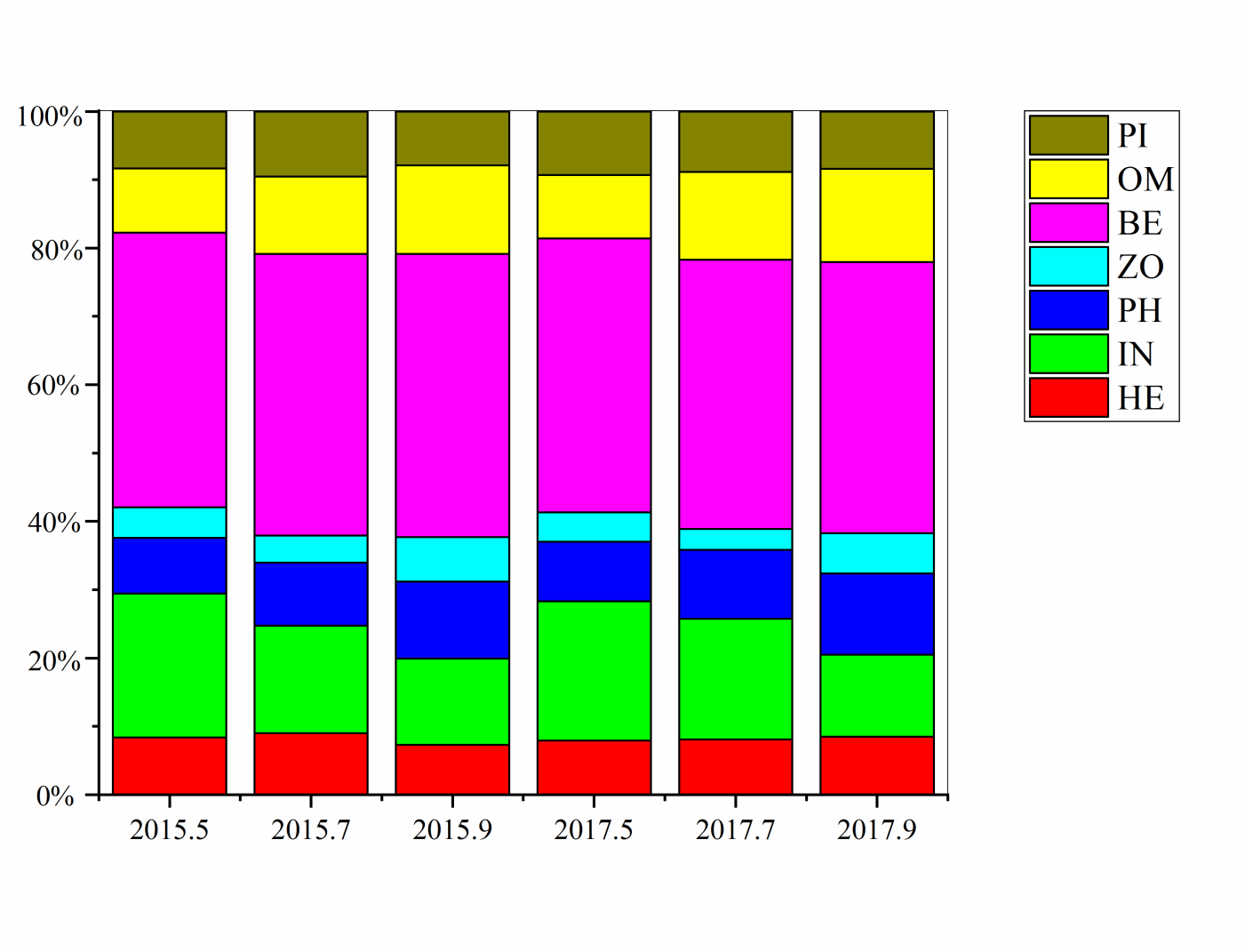


**Fig. S2** Seasonal distribution of fish trophic guilds relative biomass in Muling River Basin. Trophic guilds: aquatic plant trophic guild (herbivores, HE), aquatic insect trophic guild (insectivores, IN), phytoplanktivores trophic guild (phytoplanktivores, PH), zooplanktivores trophic guild (zooplanktivores, ZO), benthic animal trophic guild (benthivores, BE), omnivores trophic guild (omnivores, OM), piscivores trophic guild (piscivores, PI).

**Fig. S3** Seasonal and spatial distribution of fish trophic guilds biomass (g) in the Muling River basin in 2015 and 2017. Trophic guilds: aquatic plant trophic guild (herbivores, HE), aquatic insect trophic guild (insectivores, IN), phytoplanktivores trophic guild (phytoplanktivores, PH), zooplanktivores trophic guild (zooplanktivores, ZO), benthic animal trophic guild (benthivores, BE), omnivores trophic guild (omnivores, OM), piscivores trophic guild (piscivores, PI). Section: S1-S18-Upper,S19-S23-Middle, and S24-S28-Lower; Season: May (Spring), July (Summer), and September (Autumn), in 2015 and 2017, respectively. Bold means dominant fish trophic guilds (with relative biomass percentage).


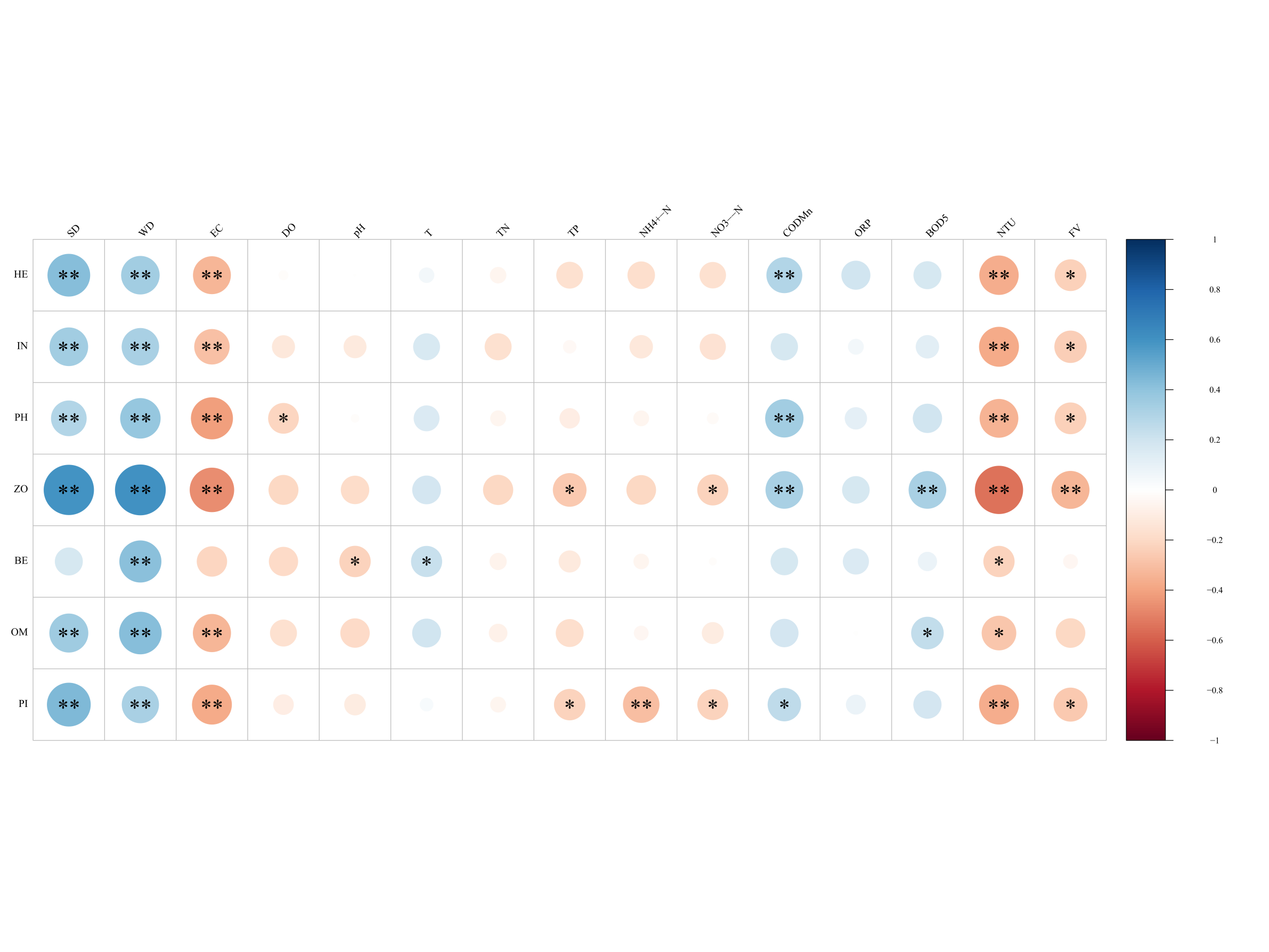


**Fig. S4** Spearman correlation analysis between fish trophic guilds and environmental factors. ^*^*P*<0.05; ^**^*P*<0.01. Environmental factors: transparency (SD, m), water depth (WD, m), electrical conductivity (EC, mS/cm), dissolved oxygen (DO, mg/L), pH value (pH), water temperature (T, ℃), total nitrogen (TN, mg/L), total phosphorus (TP, mg/L), ammonia nitrogen (NH_4_^+^-N, mg/L), nitrate nitrogen (NO_3_^-^-N, mg/L), chemical oxygen demand (COD_Mn_, mg/L), oxidation-reduction potential (ORP, mv), biochemical oxygen demand (BOD_5_, mg/L), turbidity (NTU) and flow velocity (FV, m/s). Trophic guilds: aquatic plant trophic guild (herbivores, HE), aquatic insect trophic guild (insectivores, IN), phytoplanktivores trophic guild (phytoplanktivores, PH), zooplanktivores trophic guild (zooplanktivores, ZO), benthic animal trophic guild (benthivores, BE), omnivores trophic guild (omnivores, OM), piscivores trophic guild (piscivores, PI).
